# Enhanced angiogenic potency of monocytic endothelial progenitor cells in patients with systemic sclerosis

**DOI:** 10.1186/ar3180

**Published:** 2010-11-04

**Authors:** Yukie Yamaguchi, Yuka Okazaki, Noriyuki Seta, Takashi Satoh, Kazuo Takahashi, Zenro Ikezawa, Masataka Kuwana

**Affiliations:** 1 Division of Rheumatology, Department of Internal Medicine, Keio University School of Medicine, 35 Shinanomachi, Shinjuku-ku, Tokyo 160-8582, Japan; 2 Department of Environmental Immuno-Dermatology, Yokohama City University Graduate School of Medicine, 3-9 Fukuura, Kanazawa-ku, Yokohama 236-0004, Japan

## Abstract

**Introduction:**

Microvasculopathy is one of the characteristic features in patients with systemic sclerosis (SSc), but underlying mechanisms still remain uncertain. In this study, we evaluated the potential involvement of monocytic endothelial progenitor cells (EPCs) in pathogenic processes of SSc vasculopathy, by determining their number and contribution to blood vessel formation through angiogenesis and vasculogenesis.

**Methods:**

Monocytic EPCs were enriched and enumerated using a culture of peripheral blood mononuclear cells and platelets on fibronectin in 23 patients with SSc, 22 patients with rheumatoid arthritis (RA), and 21 healthy controls. To assess the capacity of monocytic EPCs to promote vascular formation and the contribution of vasculogenesis to this process, we used an *in vitro *co-culture system with human umbilical vein endothelial cells (HUVECs) on Matrigel^® ^and an *in vivo *murine tumor neovascularization model.

**Results:**

Monocytic EPCs were significantly increased in SSc patients than in RA patients or healthy controls (*P *= 0.01 for both comparisons). Monocytic EPCs derived from SSc patients promoted tubular formation in Matrigel^® ^cultures more than those from healthy controls (*P *= 0.007). Transplantation of monocytic EPCs into immunodeficient mice resulted in promotion of tumor growth and blood vessel formation, and these properties were more prominent in SSc than healthy monocytic EPCs (*P *= 0.03 for both comparisons). In contrast, incorporation of SSc monocytic EPCs into the tubular structure was less efficient *in vitro *and *in vivo*, compared with healthy monocytic EPCs.

**Conclusions:**

SSc patients have high numbers of aberrant circulating monocytic EPCs that exert enhanced angiogenesis but are impaired in vasculogenesis. However, these cells apparently cannot overcome the anti-angiogenic environment that characterizes SSc-affected tissues.

## Introduction

Systemic sclerosis (SSc) is a multi-system connective tissue disease characterized by excessive fibrosis and microvascular abnormalities. SSc vasculopathy mainly affects small arteries, causing reduced blood flow and tissue ischemia, which leads to Raynaud's phenomenon, digital ulcers, and gangrene [1]. The pathogenesis of SSc vasculopathy is not fully understood, but several lines of evidence have shown that the primary mechanism involves enhanced vascular injury, occurring as a result of an inflammatory-immune response and ischemia-reperfusion reactions [2,3]. On the other hand, defective vascular repair machinery has recently been proposed as an alternative mechanism [4].

The formation and repair of blood vessels in adults are mediated through two different processes: angiogenesis is a process of sprouting from pre-existing vessels; it involves the proliferation and migration of mature endothelial cells. Vasculogenesis is mediated through the recruitment and *in situ *differentiation of bone marrow-derived endothelial progenitor cells (EPCs) [5]. Human EPCs, also termed circulating endothelial precursors, are progenitors lacking typical hematopoietic markers that give rise to endothelium and are characterized by a unique phenotype: positive for CD34, CD133, and vascular endothelial growth factor (VEGF) receptor type 2 [6]. We recently reported defective vasculogenesis in SSc patients, based on the reduced number of EPCs in circulation and their impaired maturation potential [7]. However, whether the number of EPCs in SSc patients is reduced or not is a matter of debate [8].

A subpopulation of circulating CD14^+ ^monocytes also has EPC-like characteristics, in terms of their expression of endothelial markers upon endothelial induction, formation of tube-like structures *in vitro*, and incorporation into newly formed blood vessels *in vivo *[9]. This EPC subset of myeloid origin, termed monocytic EPCs, is apparently distinct from "classic" EPCs [10] and may share characteristics of early outgrowth cells and circulating angiogenic cells [11]. Monocytic EPCs are now considered oligopotent cells that may differentiate into endothelium as well as into other elements of the vasculature, such as pericytes and smooth muscle cells, but their *in vivo *vasculogenic potential is far inferior to "classic" EPCs [12]. In addition, monocytic EPCs contribute to new vessel formation and vascular repair through angiogenesis by angiogenic factor secretion and other mechanisms [11,12].

We recently reported that primitive cells with the capacity to differentiate into various types of mesenchymal-lineage cells and into endothelial cells can be enriched from a subpopulation of circulating monocytes in an *in vitro *culture system [13-15]. These cultured cells, termed monocyte-derived multipotential cells, have a spindle-shaped morphology and a unique phenotype positive for CD14, CD45, CD34, and type I collagen [13]. Since these monocyte-derived cells are capable of proliferating and differentiating along the endothelial lineage *in vitro *and *in vivo *[15], it is reasonable to say that circulating precursors for monocyte-derived multipotential cells are compatible with or belong among the monocytic EPCs. In this study, we evaluated the potential involvement of monocytic EPCs in SSc vasculopathy by examining their quantity as well as their angiogenic and vasculogenic properties using the procedure to enrich circulating precursors for monocyte-derived multipotential cells.

## Materials and methods

### Patients and controls

We studied blood samples from 23 patients with SSc, 5 men and 18 women (60.2 ± 14.8 years), who fulfilled the American College of Rheumatology (ACR) preliminary classification criteria [16], and from 21 healthy controls, 4 men and 17 women (63.6 ± 10.4 years). In some analyses, samples from 22 patients with rheumatoid arthritis (RA), 2 men and 20 women (58.8 ± 8.1 years), who fulfilled the ACR classification criteria [17], were used as a disease control. Eighteen SSc patients (78%) were classified as having diffuse cutaneous SSc according to published criteria [18]. Disease duration was 11.1 ± 9.6 years, and 10 (43%) patients had received their diagnosis within five years. Clinical characteristics at the time of blood sampling are summarized in Table 1. A series of SSc-related autoantibodies were determined using indirect immunofluorescence and immunoprecipitation assays [19]. None of SSc patients received cytotoxic drug at anytime in their illness, but six patients were on low-dose corticosteroids (< 10 mg/day) at examination. All samples were obtained after the patients and control subjects gave their written informed consent in accordance with the tenets of the Declaration of Helsinki, as approved by the International Review Boards of Keio University and Yokohama City University.

**Table 1:** Clinical characteristics at the time of blood sampling in 23 patients with SSc*

Raynaud's phenomenon	23 (100%)
Digital ulcers	9 (39%)
Interstitial lung disease	10 (43%)
Current smoker	1 (4%)
Past smoker	1 (4%)
Hypertension	6 (26%)
Hypercholesterolemia	4 (17%)
Positive anti-nuclear antibody	23 (100%)
Positive anticentromere	8 (35%)
Positive anti-topoisomerase I	5 (22%)
Positive anti-U1RNP	2 (9%)
Positive anti-Th/To	3 (13%)
Positive anti-RNA polymerase III	2 (9%)

* The results are expressed as the number and frequency (%).

### Preparation and quantification of monocytic EPCs

Monocytic EPCs were enriched using a culture system we developed previously [13] with some modifications. Briefly, peripheral blood mononuclear cells (PBMCs) were isolated from heparinized peripheral blood by Lymphoprep (Fresenius Kabi Norge AS, Halden, Norway) density-gradient centrifugation. Since the number of platelets and microparticles contaminating the PBMC fraction influences the recovery of monocytic EPCs in cultures [20], PBMCs were first subjected to platelet depletion with the MACS^® ^system (Miltenyi Biotec, Bergisch Gladbach, Germany) using anti-CD61 monoclonal antibody (mAb)-coupled magnetic beads. Platelet-depleted PBMCs (3 × 10^6^) were then cultured in duplicate on fibronectin-coated six-well plates with autologous platelets (3 × 10^7^) in low-glucose Dulbecco's modified Eagle's medium supplemented with 10% fetal bovine serum (JRH Bioscience, Lenexa, KS, USA), 2 mM L-glutamine, 50 U/ml penicillin, and 50 mg/ml streptomycin (without any additional growth factors) at 37°C in a 5% CO_2 _humidified atmosphere. The medium, which contained floating cells, was exchanged for fresh medium at Day 3. At 10 days of culture, adherent cells with a spindle-like morphology were counted under an inverted microscope. The number of monocytic EPCs in 1 mL of peripheral blood was calculated as the mean of multiple measurements in proportion to the volume of peripheral blood that yielded 3 × 10^6 ^PBMCs at the isolation procedure. The expression of CD1a, CD14 CD34, CD80, CD83, and VEGF receptor type 1 (VEGFR1) and uptake of 1,1'-dioctadecyl-3,3,3',3'-tetramethylindocarbocyanine (Dil)-labeled acetylated low-density lipoprotein (acLDL) (2.5 mg/ml: Molecular Probes, Eugene, OR, USA) in monocytic EPCs was evaluated by flow cytometry, while expression of CD31, CD144, and VEGFR1 on adherent cells was evaluated by immunohistochemistry [13].

In some instances, monocytic EPCs were cultured on fibronectin-coated plastic plates for 3, 5, 7, 10, and 14 days in endothelial cell basal medium-2 (EBM-2; Clonetics, San Diego, CA, USA) supplemented with EBM-2 MV SingleQuots^® ^(Clonetics) containing 5% fetal bovine serum, VEGF, basic fibroblast growth factor, epidermal growth factor, insulin-like growth factor-1, heparin, and ascorbic acid [15]. The medium was exchanged with fresh medium every three to four days. Differentiation into mature endothelial cells was evaluated by immunohistochemistry for expression of VEGF receptor type 2 (VEGFR2) and von Willebrand factor (vWF) [15].

### *In vitro *vascular tube formation in Matrigel^® ^culture

The capacity of monocytic EPCs to promote the formation of tubular structures by mature endothelial cells was examined in Matrigel^® ^culture (BD Biosciences, San Diego, CA, USA) as described previously [15]. Briefly, a suboptimal number (10^4^) of human umbilical vein endothelial cells (HUVECs), which formed a small number of short tubular structures when cultured alone, were cultured in duplicate in EBM-2 supplemented with EBM-2 MV SingleQuots^® ^on 12-well Matrigel^® ^plates (BD Biosciences) with or without monocytic EPCs (10^4^). Each experiment was conducted by pairing samples of monocytic EPCs derived from SSc patients and from healthy controls. As a control, monocytic EPCs were cultured alone on Matrigel^®^. After 24 hours, the total tube length in each well was measured. The capacity of monocytic EPCs to enhance tubular formation was assessed as the ratio of the total tube length in the culture of HUVECs plus monocytic EPCs to the length in the culture of HUVECs alone. In some instances, culture supernatants of HUVECs (10^4^) plus monocytic EPCs (10^4^) in EBM-2 supplemented with EBM-2 MV SingleQuots^® ^on 12-well Matrigel^® ^plates were collected as conditioned medium, and used in the second Matrigel^® ^cultures with HUVECs alone (10^4^). The capacity to enhance tubular formation was assessed as the ratio of the total tube length in the culture with conditioned medium of HUVECs plus monocytic EPCs to the length in the culture with conditioned medium of HUVECs alone.

In some experiments, monocytic EPCs and HUVECs were pre-labeled with PKH67 (Sigma, St. Louis, MO, USA), and Dil-acLDL, respectively, and cultured together in Matrigel^® ^[15]. The cells were observed at 24 hours under a fluorescence microscope, and the capacity of the monocytic EPCs to be incorporated into the tubular structure was evaluated as the number of PKH67-labeled monocytic EPC-derived cells within the tubular structure divided by the total tube length (cells/mm).

### *In vivo *tumor neovascularization model

A murine tumor neovascularization model was described previously [15]. Briefly, murine colon carcinoma CT-26 cell line cells (2.5 × 10^5^) were transplanted beneath the skin of the back of severe combined immunodeficient (SCID) mice (Charles River Japan, Yokohama, Japan) in conjunction with or without monocytic EPCs (10^4 ^or 10^5^). Each experiment was conducted using a pair of monocytic EPCs derived from SSc patients and from healthy controls. Ten days later, the mice were sacrificed and the volume of the subcutaneous tumor was calculated as follows: 0.5 × longest diameter × (shortest diameter)^2^. Formalin-fixed, paraffin-embedded specimens were stained with hematoxylin and eosin. The number of blood vessels carrying erythrocytes was counted in 10 independent fields at a magnification of x10, and the results were expressed as the mean. Frozen specimens (8-mm thick) were incubated with rat anti-mouse CD31 mAb (BD Biosciences) in combination with mouse anti-human CD31 mAb-fluorescein isothiocyanate (FITC) conjugate (Chemicon, Temecula, CA, USA) or mouse anti-human leukocyte antigen (HLA) class I-FITC conjugate (Sigma), followed by incubation with AlexaFluor^®^488 anti-FITC and AlexaFluor^®^568 anti-rat-specific IgG antibodies (Molecular Probes). Negative controls were sections incubated with isotype-matched mouse or rat mAb to an irrelevant antigen, instead of the primary antibody. Nuclei were counter-stained with TO-PRO3 (Molecular Probes). These slides were examined with a confocal laser fluorescence microscope (LSM5 PASCAL; Carl-Zeiss, Göttingen, Germany). The efficiency of monocytic EPC incorporation into the vascular wall was evaluated as the proportion of blood vessels containing human CD31-expressing endothelial cells in at least 100 blood vessel sections.

### Statistical analysis

All continuous variables were expressed as the mean ± standard deviation. Comparisons between two groups were tested for statistical significance using the Mann-Whitney *U *test or Wilcoxon *t*-test as appropriate.

## Results

### Number of monocytic EPCs

**Figure 1:**
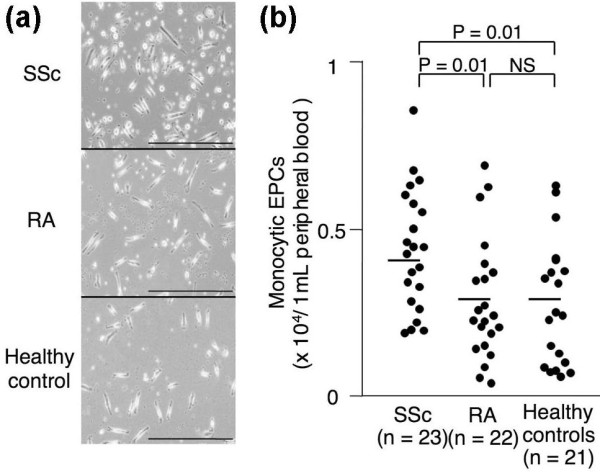
**Monocytic EPCs enriched in culture on fibronectin**. **(a) **Representative images of monocytic EPCs cultured for 10 days, from an SSc patient, an RA patient, and a healthy control. Adherent cells with a typical spindle shape are regarded as monocytic EPCs. Scale bars = 500 mm. **(b) **Monocytic EPCs were quantified in SSc patients, RA patients, and healthy controls, and expressed as the number in 1 mL of peripheral blood. Horizontal bars indicate the mean values. NS, not significant.

**Figure 2:**
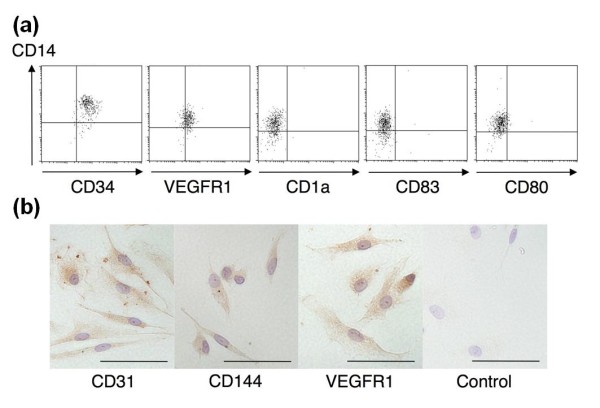
**Protein expression profiles of monocytic EPCs**. **(a) **Flow cytometric analysis of monocytic EPCs derived from a healthy control. Cells were stained with anti-CD14 mAb plus mAb to CD34, VEGFR1, CD1a, CD83, or CD80, and analyzed by flow cytometry. **(b) **Immunohistochemical analysis of monocytic EPCs. Cells were stained with a mouse mAb to the endothelial marker, as indicated. Controls were incubated with an isotype-matched mouse mAb to an irrelevant antigen. Nuclei were counterstained with hematoxylin. Bars, 50 μm.

We enriched for monocytic EPCs by culturing PBMCs on fibronectin with autologous platelets. After 10 days, adherent cells with a spindle-shaped morphology made their appearance in all cultures (Figure 1a). Nearly all adherent cells obtained in this culture were positive for both CD14 and CD34 (Figure 2a), as shown in our previous report [13]. These adherent cells expressed VEGFR1 and incorporated Dil-labeled acLDL, but lacked expression for dendritic cell markers CD1a and CD83 or a mature macrophage marker CD80 (Figure 2a). Immunohistochemistry showed expression of a series of endothelial markers, including CD31, CD144, and VEGFR1, by nearly all adherent cells (Figure 2b). These findings indicate that monocytic EPCs enriched in our culture system with fibronectin are a homogeneous cell population in terms of protein expression profiles. When the number of monocytic EPCs was compared among 23 patients with SSc, 22 with RA, and 21 healthy controls, there were significantly more monocytic EPCs in cultures derived from SSc patient samples, than in those from RA patients or healthy controls (*P *= 0.01 for both comparisons; Figure 1b). There was no significant association between the number of monocytic EPCs in culture and the SSc patients' disease duration, disease subset, digital ulcers, interstitial lung disease, SSc-related autoantibodies, or treatment with corticosteroids.

### Capacity of monocytic EPCs to promote tubule formation *in vitro*

We first evaluated potentials of monocytic EPCs to differentiate into mature endothelial cells *in vitro*. Monocytic EPCs from seven patients with SSc and seven healthy controls cultured in endothelial induction medium resulted in expression of mature endothelial cell markers VEGFR2 and vWF, and expression of VEGFR2 and vWF was observed after Days 5 and 7, respectively, in all samples irrespective of the presence or absence of SSc (data not shown).

**Figure 3:**
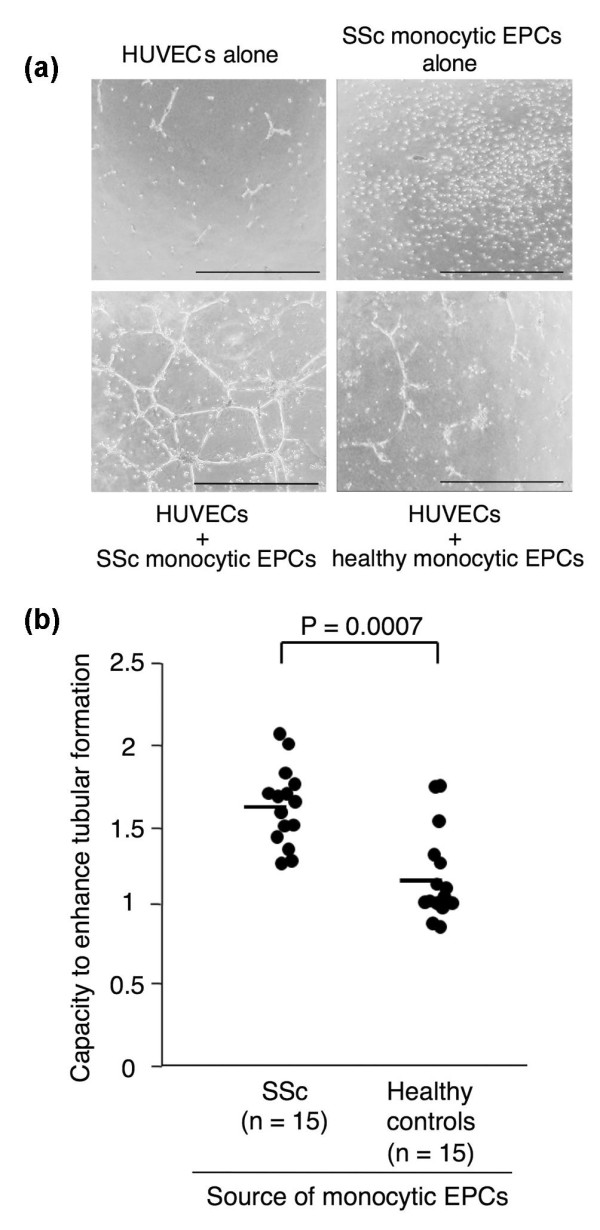
**Capacity of monocytic EPCs to promote tubular formations in co-culture with HUVECs in Matrigel^®^**. **(a) **Representative images of Matrigel^® ^cultures of HUVECs (10^4^) alone, monocytic EPCs (10^4^) from an SSc patient alone, and HUVECs (10^4^) plus monocytic EPCs (10^4^) from an SSc patient and a healthy control. Scale bars = 1 mm. **(b) **Capacity of monocytic EPCs to enhance the tubular formation was expressed as the ratio of total tube length in the culture of HUVECs plus monocytic EPCs to the length in the culture of HUVECs alone, and compared between SSc patients and healthy controls. Horizontal bars indicate the mean values.

Next, monocytic EPCs from healthy and SSc subjects cultured alone on Matrigel^® ^failed to form tubular structures, but they promoted tubule formation in 24-hour co-culture with HUVECs (Figure 3a). In this short-term culture, tube was formed mainly by cell migration. When we examined monocytic EPCs derived from 15 pairs of SSc patients and healthy controls (Figure 3b), the capacity of the monocytic EPCs to enhance tubular structure formation was significantly greater in cultures from SSc patients than in those from healthy controls (*P *= 0.0007).

**Figure 4:**
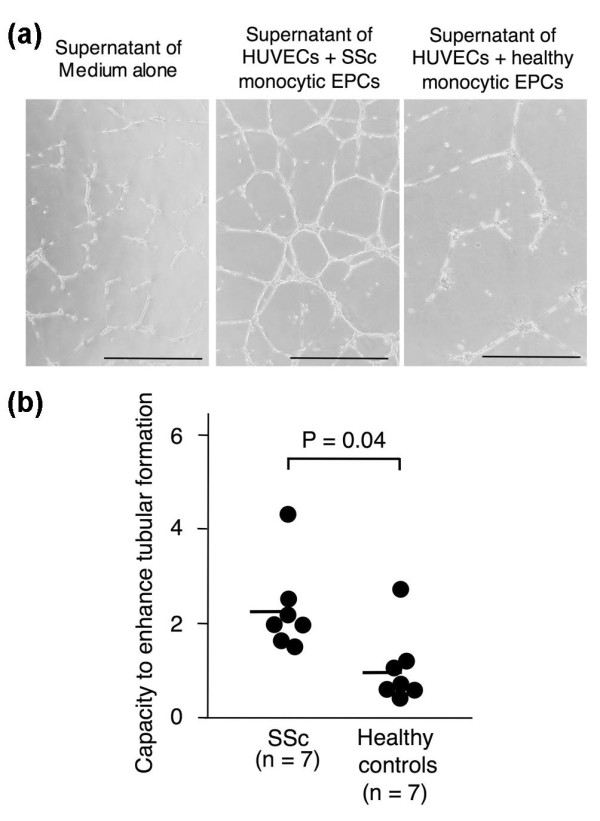
**Capacity of monocytic EPC-derived conditioned medium to promote tubular formations in culture of HUVECs in Matrigel^®^**. **(a) **Representative images of Matrigel^® ^cultures of HUVECs in the presence of culture supernatants of HUVECs (10^4^) alone, HUVECs (10^4^) plus monocytic EPCs (10^4^) from an SSc patient, and HUVECs (10^4^) plus monocytic EPCs (10^4^) from a healthy control. Scale bars = 500 μm. **(b) **Capacity to enhance tubular formation was expressed as the ratio of total tube length in the culture with conditioned medium of HUVECs plus monocytic EPCs to the length in the culture with conditioned medium of HUVECs alone, and compared between SSc patients and healthy controls. Horizontal bars indicate the mean values.

Two potential mechanisms could account for the monocytic EPCs' role in promoting tubule formation *in vitro*: the support of tubule formation by HUVECs and their own incorporation into the tubular structure. To examine whether the former mechanism was involved in this process, we collected supernatants of the Matrigel^® ^cultures as conditioned medium, and examined their capacity to promote tube formation in the second Matrigel^® ^cultures with HUVECs alone (Figure 4). The conditioned medium was prepared from the first cultures with HUVECs plus monocytic EPCs (10^4^) derived from seven pairs of SSc patients and healthy controls, and from the cultures with HUVECs alone. The capacity to enhance tubular formation was significantly greater in SSc-derived conditioned medium than in healthy control-derived medium (*P *= 0.04).

**Figure 5:**
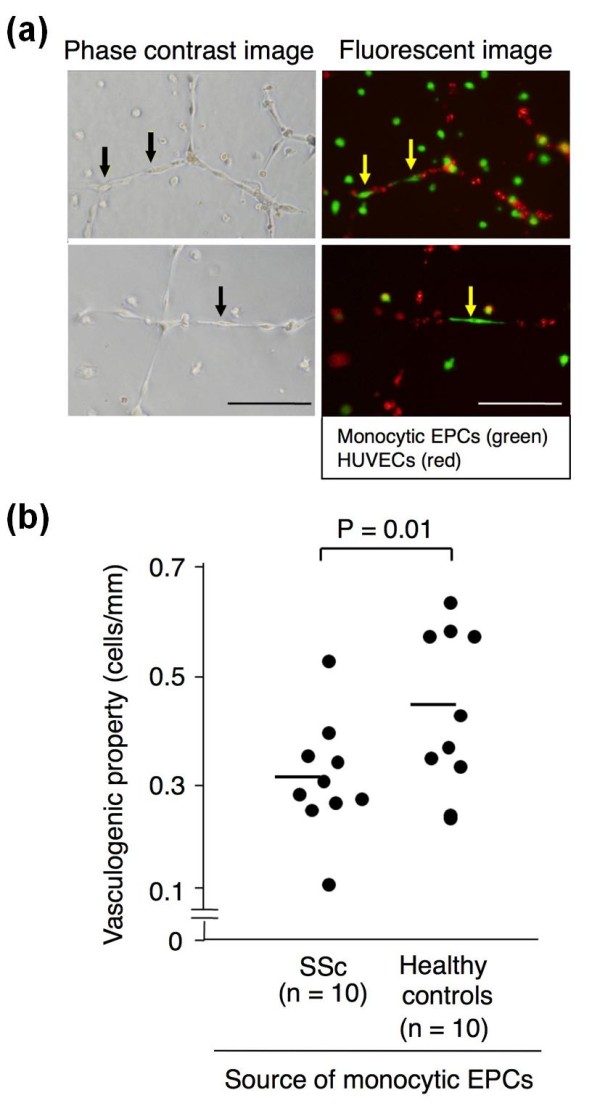
***In vitro *vasculogenic property of monocytic EPCs in Matrigel^® ^culture**. Monocytic EPCs labeled with PKH67 (green) and HUVECs labeled with Dil-acetylated LDL (red) were cultured together on Matrigel^®^. **(a) **Typical phase-contrast (left) and fluorescent (right) images of the same field of monocytic EPCs from an SSc patient (upper) and a healthy control (lower). An arrow indicates a monocytic EPC-derived cell incorporated into the tubular structure. Scale bars = 100 μm. **(b) **The vasculogenic property of monocytic EPCs was calculated as the number of monocytic EPCs within the tubular structure divided by the total tube length (cells/mm), and compared between SSc patients and healthy controls. Horizontal bars indicate the mean values.

To further evaluate the ability of monocytoc EPCs to be incorporated into the tubular structures, monocytic EPCs were pre-labeled with a green fluorescent cell linker PKH67 and cultured with Dil-acLDL-labeled HUVECs in Matrigel^®^. We found that a small number of monocytic EPCs were integrated into tubular structures that were primarily formed by HUVECs (Figure 5a). When we tested monocytic EPCs derived from 10 pairs of SSc patients and healthy controls (Figure 5b), their capacity to integrate into tubule formation was less efficient in the SSc-derived cultures than in those from healthy controls (*P *= 0.01).

### Capacity of monocytic EPCs to promote neovascularization *in vivo*

**Figure 6:**
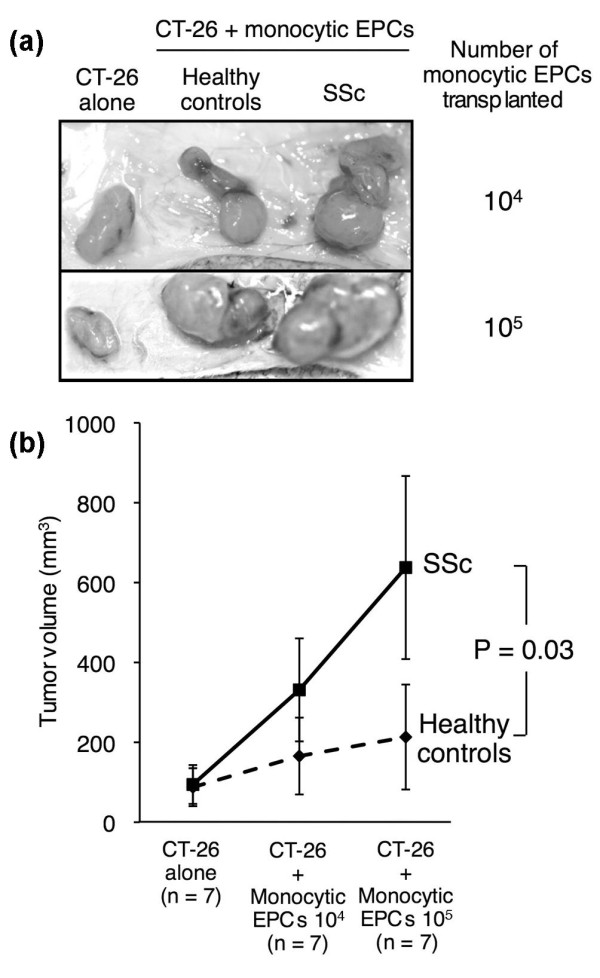
**Tumor growth after transplantation of monocytic EPCs in the *in vivo *tumor neovascularization model**. Tumors from colon carcinoma CT-26 cells injected subcutaneously into the back of mice alone or in combination with monocytic EPCs (10^4 ^or 10^5^) derived from SSc patients or healthy controls. Tumor growth was assessed 10 days later. **(a) **Representative subcutaneous tumors from mice that received transplanted CT-26 cells alone, or CT-26 cells along with monocytic EPCs (10^4 ^or 10^5^) from a healthy control or an SSc patient. **(b) **Tumor volumes in mice that received transplants of CT-26 cells alone, or CT-26 cells in combination with monocytic EPCs from SSc patients or healthy controls (10^4 ^or 10^5^). Results are shown as the mean and standard deviation.

The *in vivo *capacity of monocytic EPCs to promote blood vessel formation was evaluated using a murine tumor neovascularization model [15]. Murine colon carcinoma CT-26 cells were injected beneath the back of SCID mice, either alone or in combination with monocytic EPCs derived from SSc patients or healthy controls. As shown in Figure 6, co-transplantation of CT-26 cells with monocytic EPCs promoted tumor growth, and the amount of growth depended on the number of monocytic EPCs. A comparison of tumor size resulting from the co-transplantation of monocytic EPCs from SSc patients or healthy controls showed that the SSc-derived monocytic EPCs promoted significantly faster tumor growth (*P *= 0.03).

**Figure 7:**
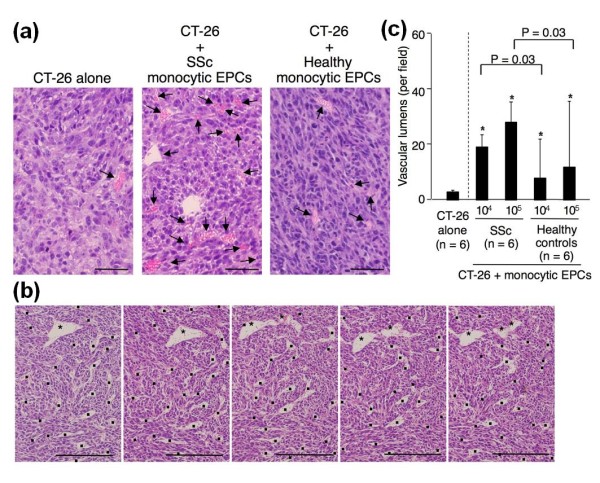
**Capacity of monocytic EPCs to promote the formation of blood vessels in a tumor neovascularization model**. **(a) **Representative tumor sections stained with hematoxylin and eosin, from mice with transplants of CT-26 cells alone or of CT-26 cells and monocytic EPCs from an SSc patient or a healthy control. Arrows indicate blood vessels carrying erythrocytes. Scale bars = 200 μm. **(b) **Representative consecutive sections of the tumor stained with hematoxylin and eosin, from mice with transplants of CT-26 cells and monocytic EPCs from an SSc patient. Asterisks indicate a relatively large blood vessel found in all consecutive sections. Dots indicate other blood vessels carrying erythrocytes. Scale bars = 500 μm. **(c) **Vascular lumen density in tumors that arose from transplanted CT-26 cells alone or CT-26 cells with monocytic EPCs (10^4 ^or 10^5^) from SSc patients or healthy controls.

A histological examination of the tumors showed that the co-transplantation of monocytic EPCs dramatically increased the number of blood vessels carrying erythrocytes compared with the transplantation of CT-26 cells alone, especially when SSc-derived monocytic EPCs were used (Figure 7a). Consecutive sections of the tumors co-transplanted with SSc-derived monocytic EPCs confirmed lateral connection of blood vessels detected as a longitudinal vessel section in the single section (Figure 7b). As shown in Figure 7c, the number of blood vessels in the tumor tissue increased with the number of transplanted monocytic EPCs, and was significantly greater in tumors that arose from the transplantation of CT-26 cells with SSc-derived monocytic EPCs than with control monocytic EPCs (*P *= 0.03 for the transplantation of 10^4 ^and 10^5 ^monocytic EPCs). Thus, the increased tumor growth caused by the presence of SSc-derived monocytic EPCs could be explained by the EPCs' enhanced ability to promote blood vessel formation *in vivo*.

**Figure 8:**
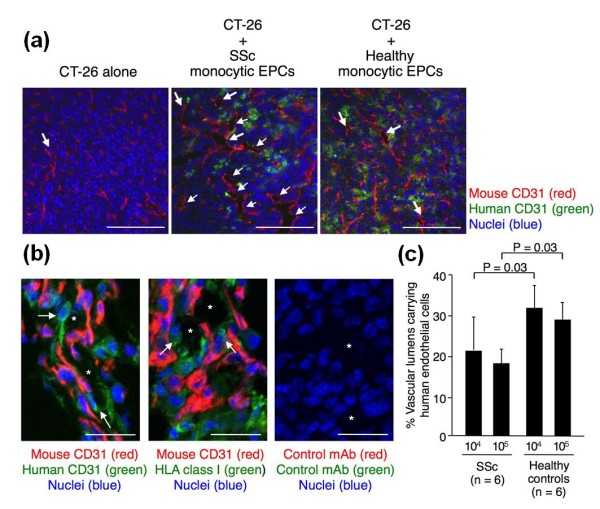
**Incorporation of monocytic EPCs into vascular lumen *in vivo *in a tumor neovascularization model**. **(a) **Representative tumor sections stained for mouse CD31 (red) and human CD31 (green), from mice with transplants of CT-26 cells alone or of CT-26 cells and monocytic EPCs from an SSc patient or a healthy control. Nuclei were counterstained with TO-PRO3 (blue). Arrows indicate blood vessels. Scale bars = 200 μm. **(b) **Representative tumor sections stained for mouse CD31 (red) and human CD31 or HLA class I (green) from mice with transplants of CT-26 cells and monocytic EPCs from a healthy control. Negative controls were sections incubated with isotype-matched mouse or rat mAb to an irrelevant antigen, instead of the primary antibody. Nuclei were counterstained with TO-PRO3 (blue). Asterisks indicate blood vessel lumen, while arrows indicate transplanted human monocytic EPCs located at the vascular wall. Scale bars = 50 μm. **(c) **The vasculogenic potency of monocytic EPCs was assessed by determining the proportion of vascular lumens carrying human CD31^+ ^endothelial cells in tumors arising from CT-26 cells co-transplanted with monocytic EPCs (10^4 ^or 10^5^) from SSc patients or healthy controls.

To further examine the contribution of vasculogenesis in this process, we evaluated the distribution of the transplanted human monocytic EPCs in the tumors by detecting cells expressing human CD31 (Figure 8a). The majority of transplanted monocytic EPCs expressing human CD31 were detected outside of the vascular lumen, but some blood vessels included cells expressing human CD31 but did not express mouse CD31 (Figure 8b). Similar findings were observed when mAb to HLA class I was used instead of anti-human CD31 mAb. When the proportion of blood vessels carrying endothelial cells expressing human CD31 was evaluated, fewer monocytic EPCs from SSc patients were incorporated into the vascular wall than monocytic EPCs from healthy controls; this difference was statistically significant when 10^4 ^and 10^5 ^monocytic EPCs were used for transplantation (*P *= 0.03 for both comparisons, Figure 8c).

## Discussion

In this study, we demonstrated that circulating monocytic EPCs were increased in the peripheral blood of SSc patients. In addition, *in vitro *and *in vivo *functional analyses revealed that monocytic EPCs derived from SSc patients had an enhanced ability to promote blood vessel formation. This characteristic was primarily attributable to an enhanced angiogenic property through production of angiogenic factors. Additional studies to identify monocytic EPC-derived soluble factors responsible for the difference in angiogenic property between SSc patients and healthy individuals are underway. In contrast, the EPCs' ability to be incorporated into vessels and differentiate into mature endothelial cells was rather impaired in SSc patients. This finding may support an early report showing that the angiogenic capacity of PBMCs from SSc patients was inferior to that of healthy controls, but when monocytes were enriched and used in the same assay system, the SSc patients' samples showed an enhanced angiogenic capacity [21].

Monocytic EPCs promote angiogenesis by secreting a variety of angiogenic factors, in a paracrine manner [12,15,22,23], and by differentiating into other elements of the vasculature, such as pericytes and smooth muscle cells, thereby contributing to the outer layers of blood vessels [11,12,24]. Skin biopsies from SSc patients were investigated for their angiogenic activity using the chick embryo chorioallantoic membrane assay [25] and the SCID mouse skin xenograft model [26], and in both studies the SSc grafts induced a prominent increase in new blood vessel formation in the surrounding normal tissue, compared with grafts from healthy subjects. Since dense mononuclear cell infiltrates were detectable around the newly formed blood vessels in those models, it is likely that SSc skin has a strong intrinsic activity that recruits angiogenic cells, such as monocytic EPCs, from the circulation, probably through chemokine production. Taken together, the robust angiogenic push observed in SSc patients may result, in part, from crosstalk between the affected tissue and circulating monocytic EPCs. In addition, capacity of circulating monocytic EPCs in SSc patients to home to the pathogenic site appears to be intact.

One of the limitations of this study is the method employed to quantify circulating monocytic EPCs, which used the short-term culture, instead of a direct analysis of freshly prepared cells. This is because of a lack of a definitive marker for monocytic EPCs in circulation. Our method is able to enrich monocytic EPCs from PBMCs by utilizing the capacity of monocytic EPCs to bind to fibronectin. Variability in the fibronectin binding capacity may influence the recovery of monocytic EPCs in the culture, but there was no difference in an expression level of α1β5 integrin, a receptor for fibronectin, between circulating CD14^+ ^monocytes from SSc patients and those from healthy individuals (data not shown). It has also been shown that weak proliferation of adherent CD14^+ ^monocytes occurs during the first 24 hours of the culture [13], and rate of proliferation was similar between SSc patients and healthy controls.

Although SSc patients have high levels of circulating monocytic EPCs with enhanced angiogenic potential, blood vessel formation is apparently insufficient in these patients [1]. This suggests the presence of mechanisms that inhibit angiogenesis at SSc-affected sites. Postnatal angiogenesis governed by endothelial cells requires a series of events, including a response to angiogenic stimuli, proliferation, the coordinated expression of proteolytic enzymes, degeneration of the extracellular matrix, and migration into the matrical space [27,28]. In this regard, microvascular endothelial cells derived from the skin of SSc patients show an overproduction of metalloproteinase-12 and the resultant impairment of urokinase-type plasminogen activator receptor signaling [29] as well as a reduction in tissue kallikreins 9, 11, and 12, which are powerful effectors of angiogenesis [30]. In addition, a recent microarray analysis of microvascular endothelial cells derived from the skin of SSc patients and controls revealed the up-regulation of genes that suppress angiogenesis and the down-regulation of genes critical to cell migration and extracellular matrix-cytoskeleton coupling, which impedes angiogenesis [31]. This anti-angiogenic environment in SSc-affected tissue might interfere with the pro-angiogenic property of monocytic EPCs. Given the defective vasculogenic capacity of monocytic EPCs as well as of "classic" EPCs [7], it seems that the final balance between blood vessel formation and repair favors the suppression of neovascularization in SSc patients.

Monocytic EPCs are recruited into the circulation in response to chemokines, such as monocyte chemoattractant protein-1 (MCP-1) [32], which are up-regulated in the affected skin of SSc patients [33,34]. In addition, endothelial cells are shown to strongly induce circulating monocytes to differentiate into EPCs under hypoxic conditions [35], which is a typical feature of SSc skin [36]. In contrast, Zhu *et al. *reported that SSc serum induces the apoptosis of circulating EPCs through up-regulation of the pro-apoptotic protein Bim, an effect mediated by the inhibition of the activation/phosphorylation of Akt [37]. Therefore, in SSc patients, it is likely that the signals that mobilize monocytic EPCs are so intense that they overcome the mechanisms that reduce the number of circulating monocytic EPCs. The presence of multiple confounding factors that affect the number of circulating monocytic EPCs may explain why their number did not correlate with any clinical characteristics of the SSc patients.

In SSc patients, functionally altered monocytic EPCs would be expected to accumulate at the affected tissue, but it is unclear how they are involved in the pathogenesis of SSc, given the strong anti-angiogenic microenvironment in the affected tissue. We previously reported that monocytic EPCs can differentiate not only into endothelial cells, but also into a variety of mesenchymal-lineage cells, including adipocytes, osteoblasts, chondrocytes, fibroblasts, and skeletal and cardiac myoblasts, when they are exposed to lineage-specific induction stimuli [13,14]. Since it has been reported that monocytes acquire the ability to produce extracellular matrix components, such as collagens, in the presence of MCP-1 [38], fibrogenic environment of the affected tissues in SSc patients may induce the differentiation of monocytic EPCs into fibroblast-like cells. Alternatively, monocytic EPCs recruited to the SSc-affected sites could be a source of soluble factors, such as MCP-1, platelet-derived growth factor, and interleukin-6, all of which accelerate fibrosis.

## Conclusions

In summary, despite insufficient vascular formation and repair in SSc patients, monocytic EPCs are paradoxically increased in the circulation and possess a prominent angiogenic potential. These functionally altered monocytic EPCs apparently cannot overcome the anti-angiogenic environment at SSc-affected sites, and may eventually be involved in other aspects of SSc pathogenesis, such as the promotion of excessive fibrosis. Further studies investigating the role of monocytic EPCs in the tissue fibrosis in SSc patients are underway.

## Abbreviations

acLDL: acetylated low-density lipoprotein; ACR: American College of Rheumatology; Dil: 1,1'-dioctadecyl-3,3,3',3'-tetramethylindocarbocyanine; EPCs: endothelial progenitor cells; FITC: fluorescein isothiocyanate; HLA: human leukocyte antigen; HUVEC: human umbilical vein endothelial cell; mAb: monoclonal antibody; MCP-1: monocyte chemoattractant protein-1; PBMC: peripheral blood mononuclear cells; RA: rheumatoid arthritis; SSc: systemic sclerosis; SCID: severe combined immunodeficient; VEGF: vascular endothelial growth factor; vWF: von Willebrand factor.

## Competing interests

The authors declare that they have no competing interests.

## Authors' contributions

YY performed the acquisition of data, and analysis and interpretation of data, and wrote the manuscript. YO, NS, and TS performed the acquisition of data. KT and ZI provided peripheral blood samples and clinical information, and performed analysis of data. MK designed the experiments, performed data analysis and interpretation, and wrote the manuscript. All authors read and approved the final manuscript.
